# Shared Genetic Etiology of Primary Dilated Cardiomyopathy and Ischemic Dilated Cardiomyopathy

**DOI:** 10.3389/fcvm.2021.752662

**Published:** 2021-11-23

**Authors:** Yang Sun, Lei Xiao, Ke Li, Hong Wang, Xiuli Song, Zongzhe Li, Chenze Li, Yanghui Chen, Shiyang Li, Jin Huang, Lun Tan, Dong Hu, Ting Yu, Rui Li, Hong Wang, Li Jin, Leming Shi, Ali J. Marian, Dao Wen Wang

**Affiliations:** ^1^Division of Cardiology, Department of Internal Medicine, Tongji Hospital, Tongji Medical College, Huazhong University of Science and Technology, Wuhan, China; ^2^Hubei Key Laboratory of Genetics and Molecular Mechanism of Cardiologic Disorders, Huazhong University of Science and Technology, Wuhan, China; ^3^Collaborative Innovation Center for Genetics and Development, School of Life Sciences, Fudan University, Shanghai, China; ^4^Center for Cardiovascular Genetics, Institute of Molecular Medicine and Department of Medicine, University of Texas Health Sciences Center at Houston, Houston, TX, United States

**Keywords:** dilated cardiomyopathy, ischemic cardiomyopathy, *TTN*, whole-exome sequencing, ACMG classification

## Abstract

**Background:** Mutations in genes encoding sarcomere and cytoskeletal proteins are major causes of primary dilated cardiomyopathy (DCM). Likewise, ischemic myocardial injury is a major cause of secondary cardiac remodeling, which, in a subset, is severe and resembles DCM. The latter is referred to as ischemic dilated cardiomyopathy (IDCM). We postulated the presence of pathogenic and likely pathogenic variants (PVs and LPVs, respectively) in genes known to cause primary DCM might predispose the heart to severe cardiac dilatation and dysfunction post myocardial ischemic injury, i.e., IDCM.

**Methods:** We performed whole-exome sequencing in 1,041 patients with primary DCM, 215 patients with IDCM, and 414 healthy controls. Indices of cardiac size and function were similar between those with primary and ischemic DCM. PVs and LPVs, including the truncating variants in 36 genes known to cause primary DCM were identified and compared among the three groups.

**Results:** Pathogenic variants and LPVs were detected in 266 individuals, comprised of 215/1,041 (20.7%) patients with DCM, 27/215 (12.6%) patients with IDCM, and 24/414 (5.8%) control individuals. PVs and LPVs in the *TTN* gene were the most common and detected in 130/1,041 (12.5%) of patients with DCM, 15/215 (7.0%) of cases with IDCM, and 10/414 (2.4%) control individuals. Of 135 *TTN*tv, 118 involved exons that were >90% spliced in. These variants were found in 120/1,041 (11.5%) of DCM patients, 6/215 (2.8%) of IDCM cases, and only in 1/414 (0.2%) of the control population (*p* < 0.001 among the three groups).

**Conclusions:** Pathogenic variants and LPVs in genes known to cause primary DCM are enriched in patients with IDCM, suggesting that such variants function as susceptibility alleles for cardiac dilatation and dysfunction in post myocardial ischemic injury. Thus, IDCM shares a partial genetic etiology with the primary DCM.

- **What is already known about this subject?**

Ischemic myocardial injury is a major cause of secondary cardiac remodeling, which, in a subset, is severe and resembles dilated cardiomyopathy (DCM) while DCM is a genetic disorder caused from mutations in genes encoding sarcomere and cytoskeletal proteins of genes.

- **What does this study add?**

Ischemic dilated cardiomyopathy (IDCM) shares a partial genetic etiology with the primary DCM.

- **How might this impact on clinical practice?**

The findings might prompt the precision diagnosis of IDCM.

## Introduction

The heart responds to a stimulus, whether internal or external, with hypertrophy, dilatation, and dysfunction. Dilated cardiomyopathy (DCM), characterized by cardiac dilatation and systolic dysfunction, represents a common response of the heart to an internal stimulus, namely, a genetic mutation. DCM is mainly caused by the pathogenic variants (PVs) in genes encoding sarcomere and cytoskeletal proteins. The *TTN* gene, encoding the giant protein titin, is the major cause of primary DCM. PVs and likely PVs (LPVs) in the *TTN* gene are found in approximately 15% of the sporadic DCM cases (1).

Coronary artery disease and its thrombotic complications, leading to myocardial infarction (MI) and ischemia, are also major causes of cardiac dilatation and dysfunction, which is commonly referred to as ischemic cardiac remodeling (2, 3). The remodeling, which represents the response of the heart to extrinsic factors, such as impaired myocardial blood flow, is variable. Whereas most patients after a bout of myocardial injury develop stable cardiac remodeling with minimal or no evidence of heart failure, a subset progresses to severe cardiac dilatation, dysfunction, and refractory heart failure. The latter group is referred to as ischemic dilated cardiomyopathy (IDCM), which has a phenotypic resemblance to that of primary DCM.

Factors that determine inter-individual variability in the response of the heart to a genetic mutation or to an ischemic injury are not well-known. The genetic backgrounds of the individuals are expected to be important determinants of the phenotypic consequence of myocardial ischemic injury, as in other forms of cardiovascular diseases, including hereditary cardiomyopathies (4–6).

Pathogenic variants in over two dozen genes are major causes of primary DCM (7). Notable among the well-established causal variants for DCM are the truncating variants in the *TTN* gene (*TTN*tv), which are the most common causes of primary DCM (1). However, *TTN*tv are also found in the general population, albeit at a much lower population frequency (1). The characteristics of *TTN*tv that cause primary DCM, as opposed to those identified in the apparently healthy individuals, are not well-known but those located in the genomic regions corresponding to the A band of the TTN proteins are more likely to be pathogenic (8). Overall, *TTN*tv are considered as susceptibility factors to eccentric cardiac remodeling in the general population and in patients with peripartum cardiomyopathy (9, 10).

Accordingly, we postulated that the PVs and LPVs in the *TTN* and other genes known to cause DCM would increase susceptibility of the heart to cardiac dilatation and dysfunction post MI. Accordingly, PVs are expected to be more prevalent in patients with IDCM as compared to the control healthy individuals. To test this hypothesis, we performed whole-exome sequencing (WES) in 215 patients with IDCM, 1,041 patients with primary DCM, and 414 healthy controls and compared the frequencies of the PVs and LPVs among the three groups.

## Methods

### Patient Population

We recruited 1,041 patients with primary DCM, 215 patients with IDCM, and 414 healthy individuals from Tongji Hospital, Wuhan, China, between July 2007 and December 2018. Primary DCM was defined as left ventricular or biventricular dilatation [left ventricular end diastolic diameter > 33 mm/m^2^ (men) or > 32 mm/m^2^ (women)] and systolic dysfunction [left ventricular ejection fraction (LVEF) <50%], in the absence of secondary causes, such as abnormal loading conditions or coronary artery disease (CAD), according to the European Society of Cardiology guidelines (11). IDCM was characterized as left ventricular or biventricular systolic dysfunction (LVEF < 50%) after MI and/or revascularization for CAD and dilatation [Left Ventricular End-Diastolic Diameter (LVEDD) > 33 mm/m^2^ (men) or >32 mm/m^2^ (women)]. CAD was defined as the presence of >75% stenosis in at least one of the three major coronary arteries, confirmed by coronary artery angiography, a history of MI, and/or revascularization. Patients with abnormal loading conditions, such as vulvar heart disease or ventricular aneurysm resulting from MI were excluded.

The control individuals were either those who underwent coronary angiography to assess possible CAD and were found to have no significant obstructive coronary lesions (*N* = 212) or were healthy subjects from the community who had no history of heart disease including CAD (*N* = 202). The investigation conforms with the principles outlined in the Declaration of Helsinki. The study was approved by the institutional review board of the Tongji Hospital. All study participants signed written informed consents. Baseline characteristic data were extracted from a centralized hospital database or collected from a review of community health care centers. All the subjects were of Han Chinese origin based on family name and self-report.

### Whole-Exome Sequencing

Genomic DNA was extracted from peripheral blood leukocytes using the TIANamp Blood DNA Kit (Tiangen, China) and quantified with Nanodrop 2,000 (Life Technology, USA). WES was performed by Berry Genomics Co. Ltd., (Beijing, China) and WuXiAppTec (Shanghai, China) using SureSelect Human All Exon version 6.0 (Agilent) and an Illumina sequencing platform in paired-end mode of 150 bp (Illumina, USA).

The WES data were processed in accordance with the best practice of The Genome Analysis Toolkit (12). In brief, the sequencing reads were aligned to the human reference genome GRCH37 (hg19) using Burrows-Wheeler Alignment Tool, followed by removal of the duplicates using Picard tools (13). The variants in each sample were called using HaplotypeCaller and consolidated across multiple samples with the GenomicsDBImport and GenotypeGVCFs tools. Finally, the VariantRecalibrator and ApplyRecalibration tools were used to filter the raw variant call set.

### Bioinformatics Analysis

The filtered variant call set was annotated using ANNOVAR (14). The key resources used for variant annotation are listed in Supplementary Table 1. PVs and LPVs were defined per ACGM guidelines (15) and variants with a minor allele frequency (MAF) of <0.001 in the East Asian population in the Genome Aggregation database (gnomAD) dataset were included for further analysis (16). The truncating variants were defined as frameshift, non-sense, and canonical splicing site variants. Population stratification analysis were performed by VCFtools (17) and plink (18) based on genomic data from the 1,000 Genome Project. To compare the distribution of the variants in the selected gene set, ggplot2, and maftools from R were used to graph the population structure and waterfall plots (19).

### Statistical Analysis

Data are presented as mean ± SD for continuous variables and as percentages for categorical variables. Fisher exact test or Pearson chi-square test of association were used to compare the population frequencies across the groups. All *p*-values are two-tailed, and those lower than the threshold *p*-value of 0.05 was considered statistically significant.

## Results

### Characteristics of the Study Population

A total of 1,670 subjects were recruited from Tongji Hospital from January 2008 through December 2018. All individuals were of Chinese Han origin (Supplementary Figure 1). The baseline characteristics of the study populations are shown in Table 1. Patients with DCM were the youngest (mean age: 55.32 years) and the control individuals were the oldest (mean age 65.49 years). There are more male subjects in the IDCM group than in the DCM group (85.6 vs. 73.5%, respectively *p* < 0.001). Likewise, diabetes mellitus was more common in the IDCM as compared to the DCM group (32.1 vs. 16.9%, *p* < 0.001). Atrial fibrillation (AF) was common in patients with DCM as compared to the IDCM group (22.4 vs. 7.4%, respectively, *p* < 0.001). Finally, the mean LVEDD was slightly greater in patients with DCM than in those with IDCM [64.72 mm (95% CI: 63.89–65.55 mm) vs. 66.65 mm (95% CI: 66.14–67.15 mm)]. All DCM and IDCM patients had severely depressed LVEF.

**Table 1:** Characteristics of the study population.

**Characteristics**	**Control**	**IDCM**	**DCM**	***P*-values**
N	414	215	1,041	
Age, Years	65.49 ± 8.88	62.94 ± 9.97	55.32 ± 13.90	<0.001
Male, *n* (%)	187 (45.2)	184 (85.6)	765 (73.5)	<0.001
BMI, Kg/m2	22.81 ± 3.05	24.74 ± 3.35	23.89 ± 4.45	<0.001
Hypertension, *n* (%)	21 (5.1)	119 (55.9)	536 (51.5)	<0.001
Diabetes, *n* (%)	1 (0.2)	69 (32.1)	176 (16.9)	<0.001
Left bundle branch block, *n* (%)	0 (0%)	15 (7.0)	108 (10.4)	<0.001
Atrial fibrillation, *n* (%)	0 (0%)	16 (7.4)	233 (22.4)	<0.001
Ventricular tachycardia, *n* (%)	0 (0%)	25 (11.6)	137 (13.2)	<0.001
LVEDD, mm	–	64.72 ± 6.20	66.65 ± 8.23	0.001
LAEDD, mm	–	47.82 ± 9.93	45.61 ± 7.74	<0.001
IVS, mm	–	9.73 ± 1.45	9.63 ± 1.51	0.381
LVPW, mm	–	9.71 ± 1.37	9.62 ± 1.40	0.369
E/e′	–	25.03 ± 16.66	23.21 ± 14.33	0.186
E/A	–	1.48 ± 0.95	1.73 ± 1.65	0.065
LVEF, %	–	32.33 ± 8.89	31.48 ± 9.87	0.24

*BMI, body mass index; IDCM, ischemic dilated cardiomyopathy; DCM, dilated cardiomyopathy; IVS, interventricular septum; LVEDD, left ventricular end diastolic diameter; LAEDD, left atrial end diastolic diameter; LVEF, left ventricular ejection fraction*.

### Quality Control Metrics

Sequencing generated an average of 12 giga bases (GB) of clean data per sample. The mean sequence coverage was more than 110× and on an average 98.74% of the target bases in each sample achieved at least 10× coverage (Supplementary Table 2). Population structure analysis confirmed the east Asian ethnicity and revealed a homogenous population structure among three groups (Supplementary Figure 1). Given the focus on genes known to cause DCM, only variants located in the 39 genes, which are well-established as causes of primary DCM, were analyzed. The selected genes were *ABCC9, ACTC1, ACTN2, ANKRD1, BAG3, CAV3, CRYAB, CSRP3, DES, DMD, DSG2, DSP, FKTN, FLNC, FXN, ILK, JPH2, LAMA4, LAMP2, LDB3, LMNA, MYBPC3, MYH6, MYH7, NEXN, PDLIM3, PLN, RBM20, SCN5A, SGCD, SYNE1, TAZ, TCAP, TNNC1, TNNI3, TNNT2, TPM1, TTN*, and *VCL*. After quality control and filtering for rare variants, a total of 2,191 variants, comprised of missense, non-sense, frameshift, and splicing variants in the above selected were identified in the entire study population (Supplementary Figure 2).

### Population Frequencies of the PVs and LPVs

**Figure 1:**
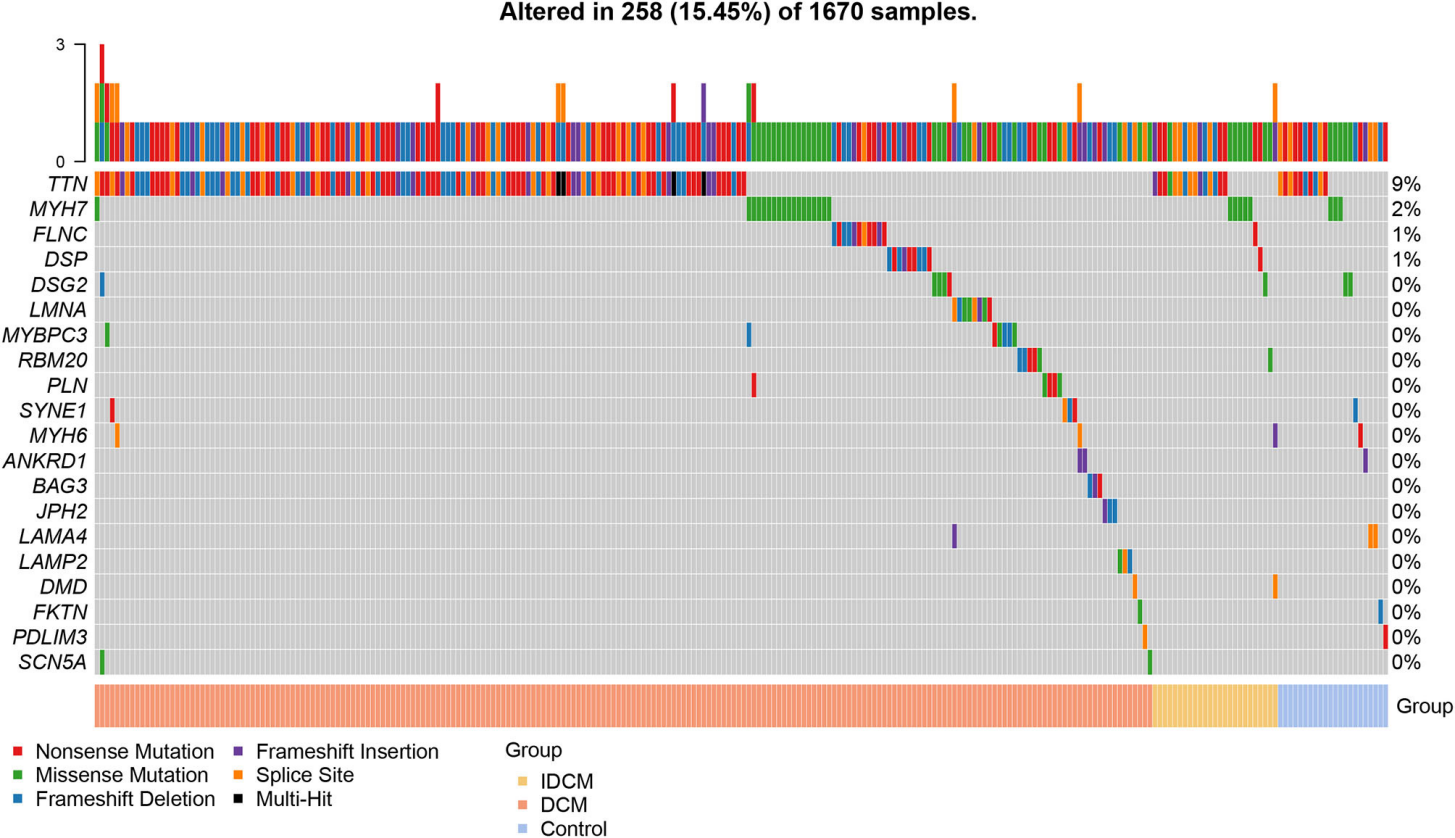
Prevalence of pathogenic and likely pathogenic variants in each cohort. The pathogenic (PVs) and likely pathogenic (LPVs) variants was defined according to American College of Medical Genetics (ACMG) guidelines. The waterfall plot shows the overall prevalence of rare pathogenic variants from 39 causal genes of dilated cardiomyopathy (DCM) in patients with DCM, patients with ischemic dilated cardiomyopathy (IDCM), and control. Among the top 20 genes, 258 individuals carried PVs or LPVs.

The number of individuals with the PVs, LPVs, and other categories of the variants in the study groups is depicted in Table 2. Population frequencies of both PVs and LPVs were the highest in DCM, intermediary in the IDCM, and the lowest in the control groups (Table 2). Overall, 215 (20.7%) patients with DCM, 27 (12.6%) patients with IDCM, and 24 (5.8%) control individuals had PVs or LPVs, indicating enrichment of the PVs and LPVs in the DCM and IDCM groups (Table 2; Figure 1).

**Table 2:** Number of individuals with each category of the pathogenic variants (PVs) and likely pathogenic variants (LPVs) per group (all genes).

**Category**	**Control**	**IDCM**	**DCM**	***P*-values**	**Pairwise P-values**		
	**(*N* = 414)**	**(*N* = 215)**	**(*N* = 1,041)**	**Three groups**	**IDCM vs. Control**	**DCM vs. Control**	**DCM vs. IDCM**
PVs, *n* (%)	5 (1.2)	7 (3.3)	74 (7.1)	<0.001	0.141	<0.001	0.052
LPVs, *n* (%)	19 (4.6)	20 (9.3)	150 (14.4)	<0.001	0.032	<0.001	0.06
PVs + LPVs, *n* (%)	24 (5.8)	27 (12.6)	215 (20.7)	<0.001	0.005	<0.001	0.008
VUS, *n* (%)	293 (70.8)	156 (72.6)	766 (73.6)	0.553	0.706	0.307	0.822
BVs + LBVs, *n* (%)	85 (20.5)	49 (22.8)	244 (23.4)	0.488	0.58	0.26	0.908

*PVs, pathogenic variants; LPVs, likely pathogenic variants; VUS, variants of uncertain significance; BVs, benign variants; LBVs, likely benign variants*.

Gene-centric analysis showed preponderance of the PVs and LPVs in the *TTN* gene, consistent with the large size of this gene, followed by the *MYH7*, which encodes major sarcomere protein myosin heavy chain 7 (Table 3). Accordingly, PVs and LPVs in the *TTN* genes were the most prevalent in all three groups with the DCM patients ranked top according to the proportion of individuals with the variants relative to the other cohorts (Table 3; Figure 1). PVs or LPVs in the *TTN* gene were detected in 130/1,041 (12.5%) of patients with DCM, 15/215 (7.0%) of cases with IDCM, and 10/414 (2.4%) control individuals (Table 3). The *TTN*tv variants were further analyzed to identify those which involved exons that were spliced in >90% in the transcripts, which are considered more likely to be pathogenic. Of the 135 *TTN*tv, 118 involved exons that were >90% spliced in and were detected in 120/1,041 (11.5%) DCM patients, 6/215 (2.8%) IDCM cases, and only 1/414 (0.2%) in the control population (*p* < 0.001 among the three groups). There are seven variants which are carried by two individuals and one variant by four individuals in DCM group.

**Table 3:** Population frequency of pathogenic and likely pathogenic variants per ACMG guideline in specific genes.

	**Control**	**IDCM**	**DCM**	***P*-values**	**Pairwise** ***P*****-values**		
**PVs + LPVs**	**(*N* = 414)**	**(*N* = 215)**	**(*N* = 1,041)**	**(Three groups)**	**IDCM vs. Control**	**DCM vs. Control**	**DCM vs. IDCM**
All genes, *n* (%)	24 (5.8)	27 (12.6)	215 (20.7)	<0.001	0.005	<0.001	0.008
*TTN, n* (%)	10 (2.4)	15 (7.0)	130 (12.5)	<0.001	0.01	<0.001	0.029
*MYH7, n* (%)	3 (0.7)	5 (2.3)	18 (1.7)	0.234	0.185	0.228	0.753
*MYBPC3, n* (%)	0 (0.0)	1 (0.5)	11 (1.1)	0.088	0.739	0.078	0.67
*ACTN2, n* (%)	0 (0.0)	1 (0.5)	9 (0.9)	0.15	0.739	0.127	0.858
*DSP, n* (%)	0 (0.0)	0 (0.0)	7 (0.7)	0.12	–	0.21	0.482
Other genes, *n* (%)	11 (2.7)	5 (2.3)	47 (4.5)	0.12	1	0.137	0.201

The missense variants in the *MYH7* gene, which considered LPVs per the American College of Medical Genetics (ACMG) guidelines, were the second most frequent variants in the study populations and were detected more commonly in DCM (18/1,041, 1.7%) and IDCM (5/215, 2.3%), as compared to the control population (3/414,0.7%), as shown in Table 3.

There were no significant differences between male or females or sex-by-genotype (PVs and LPVs) interactions in the echocardiographic indices of left ventricular size or function in patients with IDCM who carried PVs and LPVs in the *TTN* and other cardiomyopathy genes.

## Discussion

The findings of the present study, based on WES of a large number of patients with IDCM and primary DCM, showed enrichment of the PVs and LPVs in genes known to cause primary DCM in patients with IDCM, as compared to the ethnically matched control population. In accord with the large size of the *TTN* gene and the relatively large effect sizes of the truncating variants as compared to missense variants, *TTN*tv were the most enriched PVs and LPVs in the IDCM patients, followed by the PVs and LPVs in the *MYH7* gene. Overall, the prevalence of the PVs and LPVs followed a gradient being the highest in those with primary DCM, intermediary in those with IDCM, and the lowest in the control population. Collectively, the data suggest a shared genetic etiology between primary DCM and cardiac dilatation and systolic dysfunction post myocardial ischemic injury, a phenotype referred to as IDCM or ischemic heart failure.

The study benefits from a relatively large sample size of patients with IDCM and DCM and includes an ethnically-matched group, who were also sequenced as opposed to using the data on the population frequency of the variants from the existing databases, such as the gnomAD. The design of the study, in a sense, might be considered analogous to including positive control, namely the DCM cases and negative control, i.e., the control individuals. The findings of the highest prevalence of PVs and LPVs in the DCM group and the lowest in the control group offer further credence to the results. It is also important to note that both DCM and the control individuals were analyzed by WES and annotated side-by-side with the IDCM cases. Moreover, the study design also benefits from inclusion of the phenotypic extremes, i.e., those who had developed severe cardiac dilatation and dysfunction, which enables testing the hypothesis of shared genetic basis of DCM and IDCM and has a lower risk of erroneous null findings. Finally, the control population had either undergone coronary angiography and had no evidence of obstructive coronary lesions and/or had no prior history of MI and other major cardiovascular and medical diseases. The primary DCM was defined per the conventional criteria and had no obstructive coronary lesions. On the other hand, all patients with IDCM had obstructive coronary lesions and/or a history of MI, as defined by the conventional criteria. Finally, PVs and LPVs were defined per the ACGM criteria and the findings were further extended to the truncating variants, which are less amenable to spurious annotation.

The findings of the present study showing a shared genetic etiology between DCM and IDCM are in accord with the previous data showing increased population prevalence of the *TTN*tv in cases with primary sporadic DCM and peripartum cardiomyopathy (9, 10). Likewise, the findings, in principle, are in agreement with the recent data showing enrichment of the *TTN*tv and PVs or LPVs in other DCM genes in patients with heart failure, including those with heart failure due to ischemic injury (6). Collectively, the sample size of the study along with detailed phenotypic characterization and reliable categorization of the genetic variants enabled establishing the shared genetic etiology of DCM and IDCM. The higher frequency of PVs and LPVs in the DCM cases, as opposed to IDCM, likely reflects the effect sizes of the variants, those with larger effect sizes causing DCM and those with smaller ones serving as susceptibility variants requiring a second insult, such as myocardial ischemia. The notion is in accord with the gradient of effect sizes of the genetic variants (20).

The study has several limitations. The term IDCM, which may be considered a misnomer, was used to denote severe cardiac dilatation and dysfunction resulting from myocardial ischemic injury due to obstructive coronary artery disease or MI. The phenotype may also be referred to as ischemic heart failure. The study has a cross-sectional design, simply comparing the frequency of the genetic variants among the three study populations. Consequently, it has the shortcomings of the cross-sectional study design, ranging from an ascertainment to survival bias, albeit the latter is unlikely, as those with susceptibility genetic variants would be expected to have poorer survival, hence, leading to a lower frequency of such variants in the surviving individuals. A desirable study would entail a prospective longitudinal study whereby every participant undergoes genotyping (by sequencing) and serial assessment of cardiac size and function post an ischemic insult. Furthermore, the sample size of IDCM is relatively small compared to the DCM group and might weaken the significance of genetic etiology in MI population.

In conclusion, the data supports a shared genetic etiology between IDCM and primary DCM. Accordingly, the presence of the PVs and LPVs in genes known to cause DCM predisposes the heart to dilatation and dysfunction post myocardial injury. The findings imply that genetic screening could lead to identification of individuals who carrying PVs and LPVs in genes known to cause cardiomyopathies and hence, identification of those who are susceptible to developing IDCM post myocardial injury.

## Data Availability Statement

The datasets presented in this study can be found in online repositories. The names of the repository/repositories and accession number(s) can be found in the article/Supplementary Material.

## Ethics Statement

The studies involving human participants were reviewed and approved by the Institutional Review Board of the Tongji Hospital. The patients/participants provided their written informed consent to participate in this study.

## Author Contributions

DW and AM: planning of the work and funding acquisition and guarantor. YS and LX: data curation and conduct of the work. KL, HW (4th author), XS, ZL, CL, YC, SL, RL, and HW (15th author): investigation. JH, LT, TY, XS, and HW (4th author): reporting of the work. YC and DH: software. YS and YC: visualization. YS and LX: writing—original draft. DW, AM, LJ, and LS: writing—review and editing. All authors contributed to the article and approved the submitted version.

## Funding

This work was supported in part by key grants from National Nature Science Foundation of China (Nos. 81630010, 91839302, 81790624, and 8210021927), National Key R&D Program of China (No. 2017YFC0909400), and Shanghai Municipal Science and Technology Major Project (No. 2017SHZDZX01). AM was supported in part by R01 grants from NIH- National Heart, Lung, and Blood Institute (HL151737 and HL132401).

## Conflict of Interest

The authors declare that the research was conducted in the absence of any commercial or financial relationships that could be construed as a potential conflict of interest.

## Publisher's Note

All claims expressed in this article are solely those of the authors and do not necessarily represent those of their affiliated organizations, or those of the publisher, the editors and the reviewers. Any product that may be evaluated in this article, or claim that may be made by its manufacturer, is not guaranteed or endorsed by the publisher.
