# Supplementary material for: Shared Genetic Etiology of Primary Dilated Cardiomyopathy and Ischemic Dilated Cardiomyopathy
Source: Front Cardiovasc Med. 2021 Nov 23;8:752662. doi: 10.3389/fcvm.2021.752662 (PMC10236477; doi:10.3389/fcvm.2021.752662)
Supplement: Supplementary file 1 [file Data_Sheet_1.docx]

Supplementary Appendix

Supplement to: Yang Sun, et al. **Shared Genetic Etiology of Primary Dilated Cardiomyopathy and Ischemic Dilated Cardiomyopathy**

Yang Sun^1, 2^, MD; Lei Xiao^1, 2^, MD; Ke Li^1, 2,^ MD; Hong Wang^1, 2^, PhD; Xiuli Song^1, 2^, BN, Zongzhe Li^1, 2^, Chenze Li^1, 2^, MD; Yanghui Chen^1, 2^, MD; Shiyang Li^1, 2^, MD; Jin Huang^1, 2^, MD, PhD; Lun Tan^1, 2^, MD, PhD; Dong Hu^1, 2^, MD; Ting Yu^1^, BN; Rui Li^1, 2^, MD, PhD; Hong Wang^1^, MD, PhD; Li Jin^1,2,3^, PhD; Leming Shi^3^, PhD, Ali J Marian^4,*^, MD and Dao Wen Wang^1, 2, 3, *^, MD, PhD

^1^Division of Cardiology, Department of Internal Medicine, Tongji Hospital, Tongji Medical College, Huazhong University of Science and Technology, Wuhan, PR China;

^2^Hubei Key Laboratory of Genetics and Molecular Mechanism of Cardiologic Disorders, Huazhong University of Science and Technology, Wuhan 430030, PR China;

^3^Collaborative Innovation Center for Genetics and Development, School of Life Sciences, Fudan University, Shanghai, China;

^4^ Center for Cardiovascular Genetics, Institute of Molecular Medicine and Department of Medicine, University of Texas Health Sciences Center at Houston, Houston, TX 77030

Yang Sun and Lei Xiao contributed equally to this work.

**Table of contents**

**Figure S1. Population structure analysis of the study cohort.**

**Figure S2. Summary plot of rare variants from 39 well-established causal genes of DCM.**

**Table S1. Summary of exome sequencing details.**

**Table S2. Key resources used for variant annotation.**

**Figure S1. Population structure analysis of the study cohort.** The Common variants (MAF > 0.05) were used for analysis of multidimensional scaling (MDS) and principle components (PC). Multidimensional scaling confirmed the east Asian origin (A) and PC analysis (B) revealed a homogenous population structure of the of the study population. IDCM, ischemic dilated cardiomyopathy; DCM, dilated cardiomyopathy.


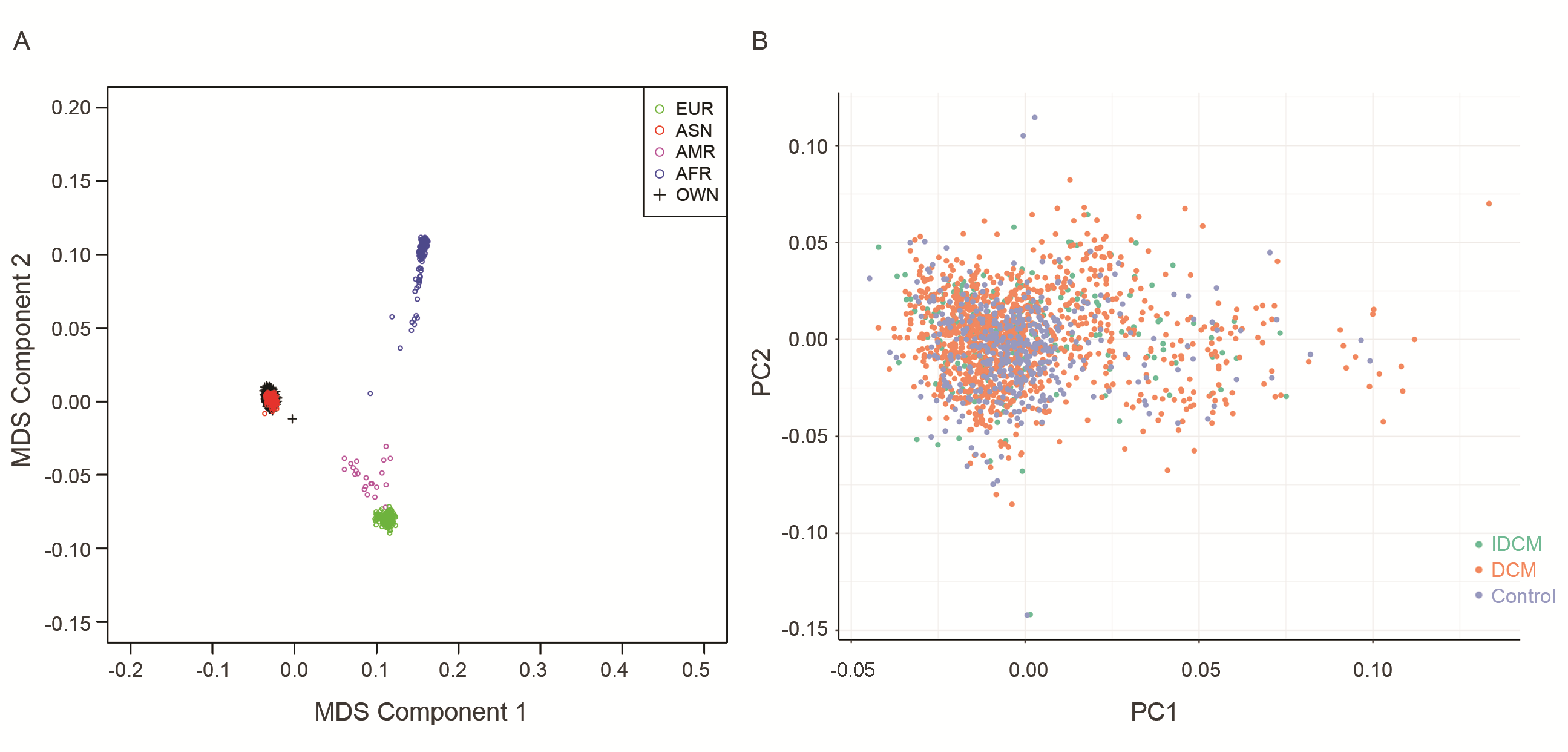

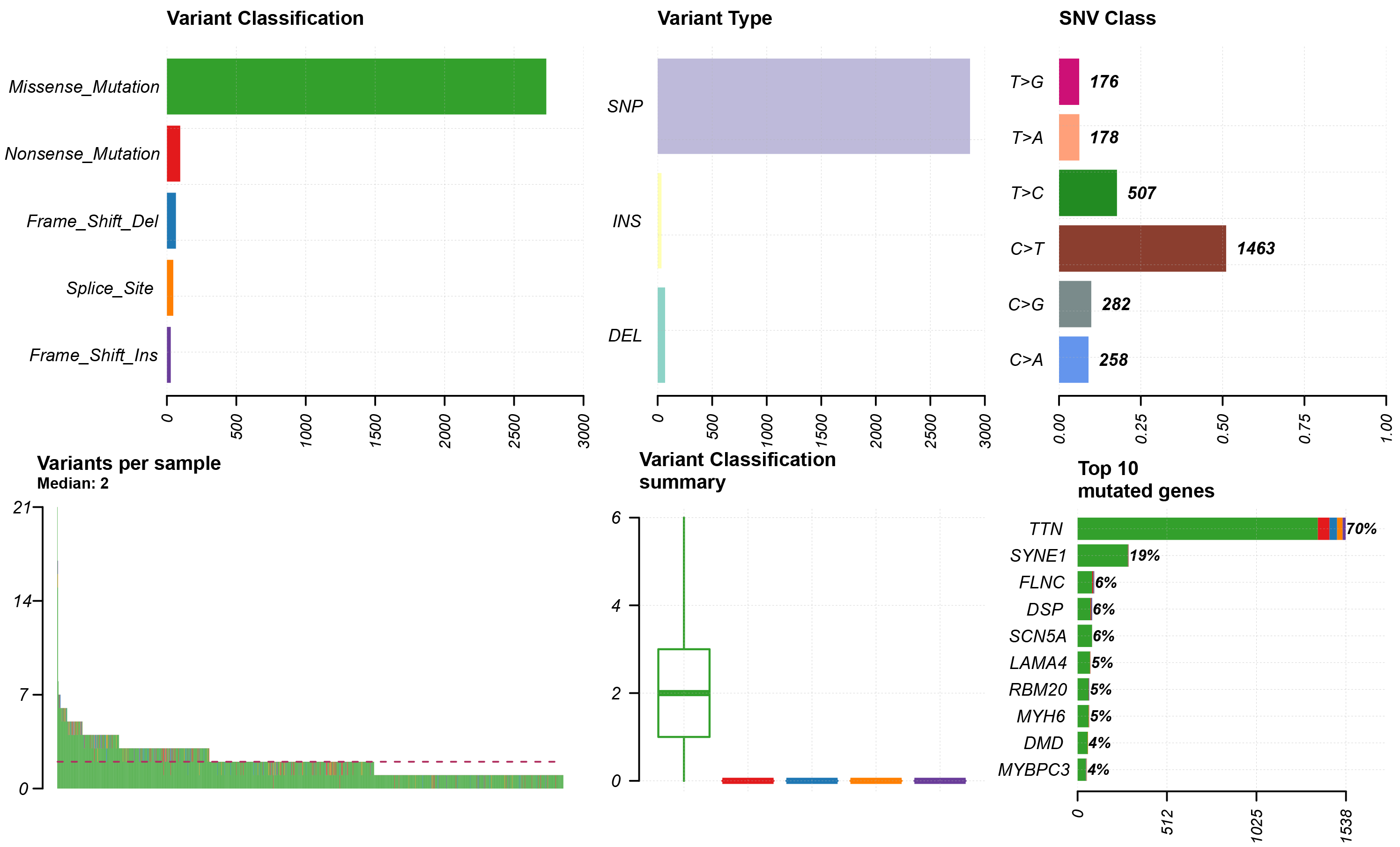


**Figure S2. Summary plot of rare variants from 39 well-established causal genes of DCM.** The summary plot was generated by maftools^1^. A total of 2,191 rare variants from the 39 genes were identified. According to the mutational frequency, *TTN* ranked top of all genes.

**Table S1. Key resources used for variant annotation.**

| **Purpose** | **Resource** | **URL,** |
| --- | --- | --- |
| Predict effect of DNA sequence variant on function of the encoded protein | ANNOVAR | http://annovar.openbioinformatics.org/ |
| Assess variant frequency (minor allele frequency, MAF) in the general population | gnomAD exome collection | http://gnomad.broadinstitute.org/about |
|  | 1000 Genomes Project | ftp://ftp.1000genomes.ebi.ac.uk |
| Assess pathogenicity of variants in disease cases | InterVar (release 01/18/2018) | http://wintervar.wglab.org/ |
|  | ClinVar (release 09/19/2021) | https://www.ncbi.nlm.nih.gov/clinvar/ |

**Table S2.** **Summary of exome sequencing details.**

| groups | Total sequence (reads, Mb) | Mapped reads (%) | Mean Read depth | % of exome > 10× | % of exome > 20× |
| --- | --- | --- | --- | --- | --- |
| DCM | 106.18 | 99.78 | 118.79 | 99.50 | 97.10 |
| DICM | 102.98 | 97.18 | 113.89 | 98.33 | 97.01 |
| Control | 101.58 | 96.41 | 115.15 | 97.05 | 95.75 |

DCM, dilated cardiomyopathy; DICM, dilated ischemic cardiomyopathy.

References:

1. Mayakonda, A., Lin, D.C., Assenov, Y., Plass, C. and Koeffler, H.P. (2018) Maftools: efficient and comprehensive analysis of somatic variants in cancer. *Genome research*, **28**, 1747-1756.
